# Oxaliplatin-induced porto-sinusoidal vascular disease manifesting as recurrent gastroesophageal variceal hemorrhage: a case report

**DOI:** 10.3389/fmed.2026.1772653

**Published:** 2026-02-19

**Authors:** Ruiqi Zhao, Chang Liang, Zhuoqing Zhuang, Yuan Xia, Junzhu Lu, Yan Zhang

**Affiliations:** Department of Gastroenterology, West China Hospital, Sichuan University, Chengdu, Sichuan, China

**Keywords:** hematemesis, hematochezia, non-cirrhotic portal hypertension, oxaliplatin, porto-sinusoidal vascular disease, thrombosis

## Abstract

Porto-sinusoidal vascular disease (PSVD) is an increasingly recognized cause of non-cirrhotic portal hypertension, characterized by structural abnormalities of the portal microvasculature and hepatic sinusoids in the absence of cirrhosis. Recognized predisposing factors include immune-mediated conditions, infections and exposure to hepatic sinusoid-toxic agents, among others. We report a 44-year-old woman with a history of oxaliplatin-based chemotherapy for colon cancer who presented with recurrent hematochezia, hematemesis, and transient encephalopathy. Evaluation demonstrated portal hypertension and portal vein thrombosis; however, liver biopsy showed no cirrhosis and the hepatic venous pressure gradient (HVPG) was within normal limits. Her medication history, combined with these findings, confirmed the diagnosis of oxaliplatin-induced PSVD. This case highlights the importance of considering PSVD in patients with non-cirrhotic portal hypertension, particularly when there is a history of exposure to agents toxic to the hepatic sinusoids, such as oxaliplatin.

## Introduction

Porto-sinusoidal vascular disease (PSVD) is a non-cirrhotic hepatic vascular disorder defined by characteristic histological alterations of the portal tracts and sinusoids. Unlike the previous concept of idiopathic non-cirrhotic portal hypertension, which required portal hypertension for diagnosis, PSVD adopts more inclusive criteria by eliminating this requirement (1). The diagnosis requires histological exclusion of cirrhosis and fulfillment of at least one of the following criteria: presence of specific pathological features; presence of specific clinical manifestations of portal hypertension; or concurrent presence of non-specific histological changes and clinical symptoms (2). Its key pathological characteristics include portal vein luminal narrowing, nodular regenerative hyperplasia (NRH), and incomplete septal fibrosis (ISF) (1), often accompanied by non-specific changes such as herniation of portal branches into the hepatic parenchyma (3), hepatic sinusoidal dilation, perisinusoidal fibrosis, and increased number or dilation of central veins or sublobular veins (4). Recognized risk factors include, in addition to immune dysfunction and genetic predisposition (5, 6), medication-induced injury caused by hepatic sinusoid-toxic agents such as oxaliplatin (7). This case describes a 44-year-old woman who developed PSVD following oxaliplatin-based chemotherapy. She presented with hematochezia, hematemesis, and altered mental status. We discuss the diagnostic challenges, imaging and histopathological findings, and management strategies, with the aim of enhancing clinician’s awareness, diagnostic accuracy, and therapeutic approach to drug-induced PSVD.

## Case presentation

A 44-year-old woman presented with a 1-year history of recurrent hematochezia, followed by a 1-month history of hematemesis and altered mental status. One year prior to admission, she developed melena that progressed to fresh bloody stools, with an estimated total blood loss of 1,000 mL. This episode was accompanied by hypotension, with a systolic blood pressure nadir of 50 mmHg. Initial gastroscopy at an external hospital revealed gastroesophageal varices. One month before admission, she presented with hematemesis and periumbilical abdominal colic. Endoscopy confirmed esophageal variceal bleeding, and she subsequently underwent endoscopic variceal ligation. Postoperatively, she developed neurological symptoms, including memory loss, slowed responsiveness, incoherent speech, and transient facial twitching. Past medical history was significant for a resection of stage pT4aN1bMx colon cancer 4 years prior, followed by adjuvant chemotherapy. On admission, the patient was conscious but fatigued. Physical examination showed no signs of chronic liver disease (e.g., palmar erythema, spider angiomas), and her abdomen was soft and non-tender.

Laboratory tests revealed pancytopenia: hemoglobin 92 g/L (ref. 115–150 g/L), platelets 78 × 10^9^/L (ref. 125–350 × 10^9^/L), and leukocytes 2.89 × 10^9^/L (ref. 3.5–9.5 × 10^9^/L). The immature reticulocyte fraction was elevated (14.9%; ref. 0.2%–2.0%), with microcytic hypochromic anemia (mean corpuscular volume 76 fL). Coagulation studies demonstrated an elevated D-dimer (3.31 mg/L FEU; ref. <0.5 mg/L FEU) and hypofibrinogenemia (1.75 g/L; ref. 2.0–4.0 g/L). Notably, liver enzymes, bilirubin, and ammonia levels were normal. Thrombophilia screening revealed reduced protein C activity (61.6%; ref. 70%–140%) and antithrombin III activity (71.5%; ref. 80%–120%).

To investigate pancytopenia and altered mental status, bone marrow biopsy and lumbar puncture were performed. Bone marrow biopsy showed hypercellularity without dysplasia or blasts; JAK2 V617F, CALR, and MPL mutations were negative. Cerebrospinal fluid analysis revealed mildly elevated protein (0.61 g/L; ref. 0.15–0.45 g/L), with negative autoimmune encephalitis-related antibodies and paraneoplastic antibodies. Autoimmune disorders were excluded based on unremarkable ANA, ENA, ANCA, and antiphospholipid antibody results.

**FIGURE 1:**
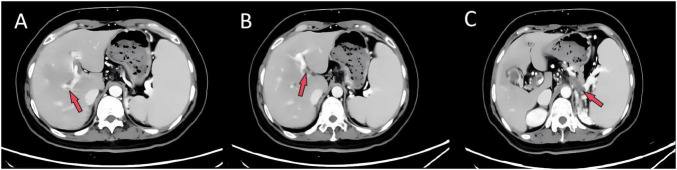
Contrast-enhanced abdominal CT. **(A)** Right portal vein branch, **(B)** left portal vein branch, and **(C)** splenic vein all reveal filling defects, suggesting widespread thrombosis.

**FIGURE 2:**
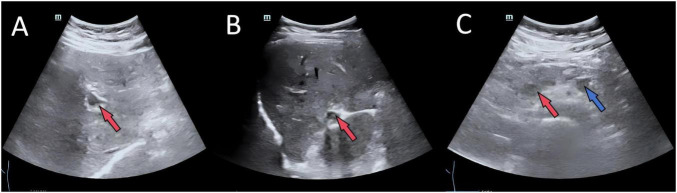
Doppler ultrasound examination confirmed the presence of partial thrombosis within the left portal vein branch **(A)**, the right portal vein branch **(B)**, the superior mesenteric vein [**(C)**, red arrow], and the splenic vein [**(C)**, blue arrow].

**FIGURE 3:**
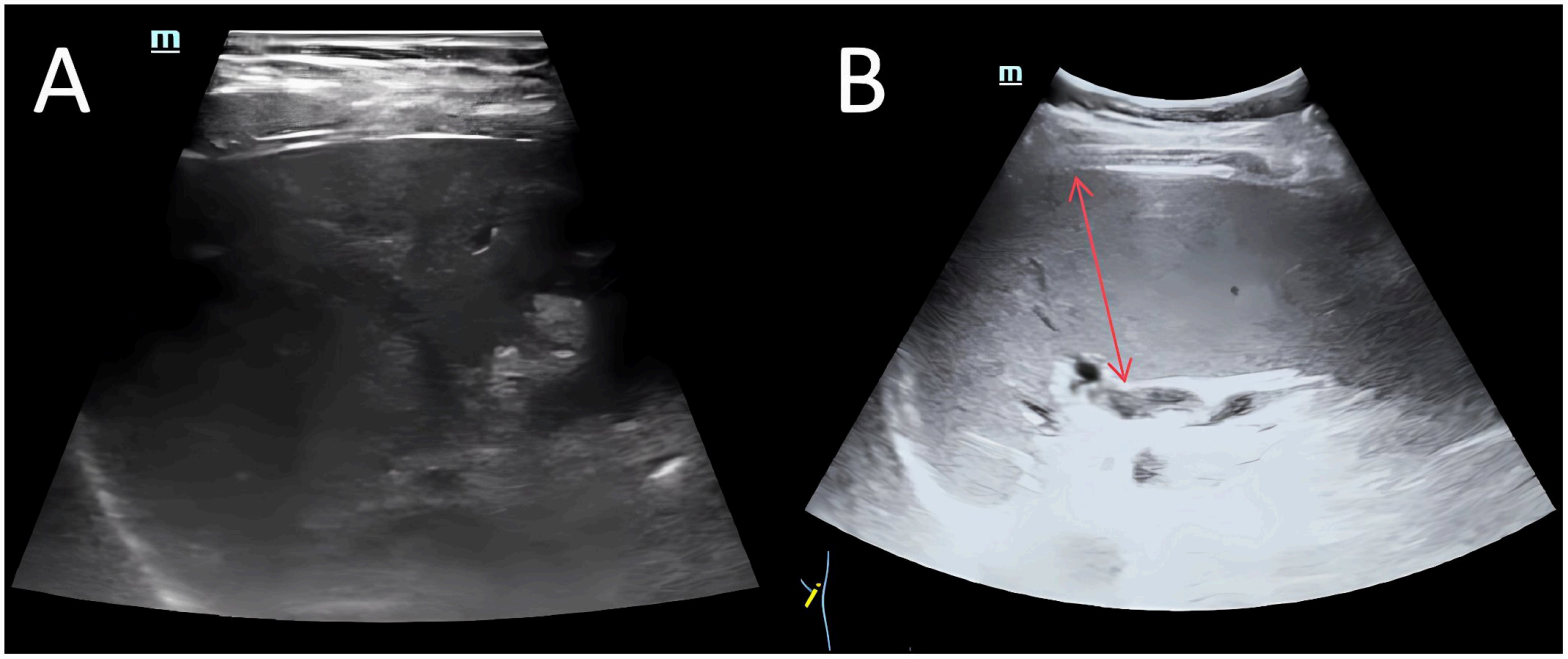
Abdominal ultrasound examination images. **(A)** Shows coarsened and enhanced hepatic parenchymal echogenicity with uneven distribution, suggesting hepatic parenchymal injury. **(B)** Demonstrates a splenic intercostal thickness greater than 4 cm, indicating splenomegaly.

Imaging studies were critical for diagnosis. Contrast-enhanced abdominal CT revealed extensive portal venous system filling defects and collateral circulation (Figure 1). Doppler ultrasonography confirmed partial thrombosis in the main portal vein, its branches, the superior mesenteric vein, and splenic vein (Figure 2). Abdominal ultrasonography showed hepatic parenchymal injury and splenomegaly (Figure 3); Vibration-Controlled Transient Elastography (FibroScan) measured liver stiffness at 11.7 kPa. PET-CT excluded malignancy, and brain MRI showed no acute pathological changes.

**FIGURE 4:**
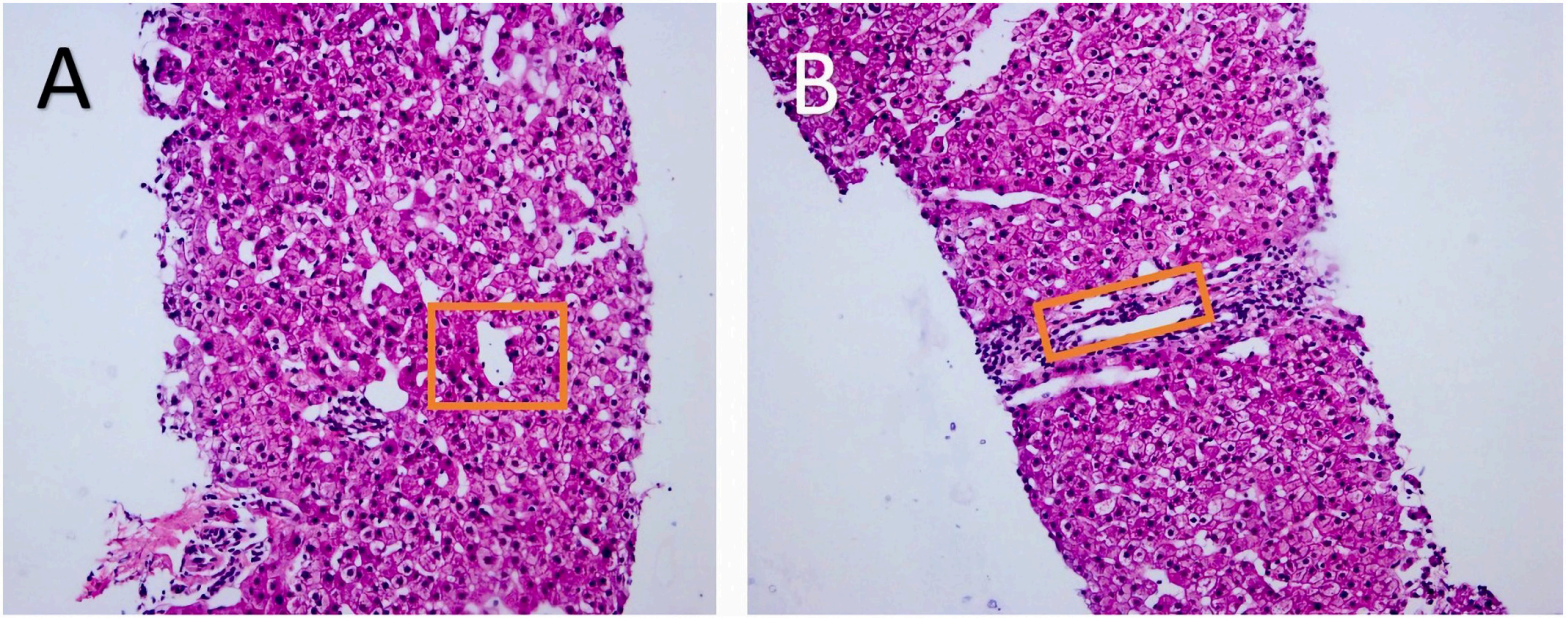
Histopathology of liver biopsy (Hematoxylin and Eosin staining). Biopsy specimens (three cores, 0.7–1.5 cm in length) containing 12 portal tracts were examined under 100 × magnification. **(A)** Shows dilation of the central vein with protrusion into the hepatic parenchyma. **(B)** Reveals hyperplasia, branching, and dilation of the interlobular veins. The aforementioned pathological changes may be observed in porto-sinusoidal vascular disease (PSVD).

To determine the etiology of portal hypertension and portal vein thrombosis, liver biopsy and hepatic venous pressure gradient (HVPG) measurement were performed in consultation with the liver intervention team. The HVPG was normal at 5 mmHg, confirming a pre-sinusoidal origin of portal hypertension. An adequate liver biopsy specimen was obtained (three cores, aggregate length > 20 mm, containing 12 portal tracts). Liver biopsy histopathology revealed interlobular vein proliferation and branching, fibrous tissue proliferation with portal tract expansion, and central vein dilatation with parenchymal herniation (Figure 4). These features, notably the absence of characteristic findings of interface hepatitis and cirrhotic nodules, combined with the imaging and clinical context, further support the diagnosis of PSVD and help to exclude autoimmune hepatitis and cirrhosis.

Further review of the patient’s medical history and external hospital records confirmed a history of oxaliplatin exposure during colon cancer treatment. Integrating this exposure with portal hypertension manifestations and definitive liver pathology, the final diagnosis was PSVD secondary to oxaliplatin-induced vascular injury. Transient altered mental status was attributed to metabolic encephalopathy, likely caused by blood loss and electrolyte imbalances—supported by the unremarkable intracranial evaluation.

During hospitalization, the patient underwent a second session of endoscopic variceal ligation to prevent variceal rebleeding. Rivaroxaban was administered as anticoagulation therapy, and carvedilol was prescribed for portal hypertension management. These interventions were continued during hospitalization and at discharge. Follow-up appointments were scheduled with the vascular surgery and gastroenterology clinics. At the most recent follow-up, 1 year after hospitalization, it was reported that the patient had undergone a third session of variceal ligation at a local hospital to prevent bleeding. However, the patient had recently self-discontinued carvedilol. Since discharge, there has been no recurrence of gastrointestinal bleeding, and the patient’s mental state and daily functioning have significantly improved. The importance of adhering to regular clinical follow-up for appropriate medication management was re-emphasized.

## Discussion

Porto-sinusoidal vascular disease (PSVD) is a non-cirrhotic vascular liver disorder characterized by portal hypertension. Its primary pathological changes affect the portal venules and hepatic sinusoids (1). The prevalence of PSVD varies geographically, accounting for 15%–34% of portal hypertension cases in developing countries (8). Known etiological factors include immune disorders, infections, certain medications such as azathioprine and oxaliplatin, toxins, genetic predisposition, and thrombophilia (9, 10).

Clinically, PSVD often presents subtly. Many patients are asymptomatic in the early stages, with splenomegaly and thrombocytopenia typically detected incidentally (11). In 20%–40% of cases, variceal bleeding is the first noticeable symptom (12). As seen in our patient, individuals with PSVD-related portal hypertension usually maintain normal or near-normal liver function, while manifestations of portal hypertension, particularly esophageal variceal bleeding, are prominent. This dissociation between preserved liver function and significant portal hypertension is a key clinical clue pointing toward PSVD (13).

Our case represents a typical but diagnostically challenging example of PSVD. The patient exhibited classic signs of portal hypertension, including gastroesophageal variceal bleeding, splenomegaly, and thrombocytopenia. Importantly, cirrhosis was confidently excluded based on the absence of fibrosis on liver biopsy, a normal hepatic venous pressure gradient, and no evidence of cirrhotic nodules on imaging. Histopathological findings, including interlobular venous hyperplasia and central vein herniation into the hepatic parenchyma, were consistent with the nonspecific but characteristic features of PSVD.

A critical diagnostic clue was the patient’s history of oxaliplatin-based chemotherapy for colon cancer. Oxaliplatin is a well-established cause of hepatic sinusoidal endothelial injury. Proposed mechanisms include increased endothelial cell porosity, oxidative stress from free radical release and glutathione depletion leading to incomplete septal fibrosis, and chronic centrilobular hypoxia that may promote nodular regenerative hyperplasia. These processes can culminate in sinusoidal obstruction syndrome and eventually evolve into PSVD (7).

The thrombotic mechanisms underlying PSVD are multifactorial, involving prothrombotic states, endothelial injury, immune dysregulation, and genetic factors (14, 15). Protein C deficiency is relatively common in these patients, reflecting an underlying thrombotic tendency (16). Endothelial damage triggered by autoantibodies such as anti-endothelial cell antibodies or by drugs like oxaliplatin can initiate microthrombus formation in the hepatic sinusoids. In this case, the combination of oxaliplatin exposure and coexisting deficiencies in both protein C and antithrombin III likely created a pronounced prothrombotic environment that directly contributed to vascular injury and thrombosis.

Management followed established principles for PSVD with portal hypertension (17). The patient received endoscopic variceal ligation and carvedilol, a combined alpha- and beta-blocker, for secondary prophylaxis against rebleeding. For patients who fail medical and endoscopic therapy, transjugular intrahepatic portosystemic shunt (TIPS) may be considered. Splenectomy or partial splenic embolization are options in cases of severe hypersplenism (18). Given the presence of extensive portal vein thrombosis and confirmed acquired thrombophilia, long-term anticoagulation with rivaroxaban was initiated. However, it should be noted that robust evidence from randomized controlled trials supporting routine prophylactic anticoagulation in PSVD remains lacking, and further clinical studies are needed to define its risks and benefits (19, 20).

## Conclusion

This case highlights the importance of recognizing Porto-sinusoidal vascular disease (PSVD) in patients with portal hypertension who lack cirrhosis. A thorough medication history, particularly exposure to chemotherapeutic agents like oxaliplatin, is critical for accurate diagnosis. Prompt identification of PSVD enables timely interventions, including discontinuation of causative agents, prevention of variceal rebleeding, and initiation of anticoagulation to prevent thrombosis formation. These findings underscore the need for heightened clinical awareness of drug-induced PSVD to improve outcomes in non-cirrhotic portal hypertension.

## Data Availability

The original contributions presented in this study are included in this article/supplementary material, further inquiries can be directed to the corresponding author.
